# Annotations

**Published:** 1888-12-01

**Authors:** 


### Annotations.

**Hot Bottles in Bed.**

Some people have a peculiar dread of warming the bed, which is partly moral and partly, as they suppose, physiological. It seems to them a sinful indulgence of the flesh, or, at best, a weakness of which they ought to be ashamed. With regard to the physiological aspect of the question their ideas are more hazy. They have an uneasy feeling that a hot bottle somehow or other "tenders" the feet, and is not conducive to general robustness. Now, all these fears may be promptly dismissed. Cold feet, the indicators of hot bottles, tend both to impair the morals and to disorder the health. They impair the morals by the discomfort they produce and the consequent loss of temper. They injure the health by diminishing the activity of the circulation and lowering the temperature of the blood. They prevent sleep, and so worry the nervous system. A hot bottle is a very desirable winter bedfellow; much better than too many bedclothes. Of course a little sense is required in the using of it. To begin with, it should be really hot, and not half cold, as some servants will persist in making it. It should be placed about the middle of the bed half an hour or three-quarters before bedtime. Then it should be pushed down to the bottom, and left there undisturbed to radiate its heat during the night. It is as well not to place the feet in actual contact with the bottle, unless they are exceedingly cold, but to put them within the radius of its warmth. It is better if the bottle have one or two folds of flannel round it. The feet are thereby protected from being burnt, and the heat is retained much longer. Hot bottles should never be placed in contact with the skin of paralysed or partially paralysed persons. Such persons often have no power of feeling, and are very liable to be burnt. In every case where a bottle is put into a child's bed, it should be carefully wrapped in flannel. A hot bottle is undoubtedly a great comfort in cold weather, and is as safe and beneficial as it is comfortable.

**The Doctor and his Fee.**

Doctors have proverbially a great regard for fees. Most of the proverbs on this point would, however, bear a good deal of modification. Everybody knows that medical men do a vast amount of work without fees, and do it cheerfully, and without grumbling. The Duke of Abercorn, who a few days ago presided at a meeting of the Society of Arts for the Promotion of Irish Industries, told a good story of a too "cute" physician who was hoist with his own petard. Off Donegal is a small island called Tory Island. In this favoured spot the inhabitants are said to be unreasonably robust. They never need a doctor, and they never die. That, at any rate, is the rule, to which, however, an occasional exception has sometimes to be recorded. There is no resident doctor on the island at all. If any medical attendance is wanted it has to be sought on the mainland. The nearest doctor, who presumably is so happy as to have no rival near, is, it seems, rather hard at a bargain. Recently a lady of Tory Island took it into her head that she would like to see the man of physic. With a want of gallantry unworthy of a physician and an Irishman he, on being sent for, demanded his fee in advance. Not only his fee, but a good fee: a guinea, and nothing less. There seems to have been some little difficulty about the payment, but eventually the business was arranged, and Æsculapius took ship, or rather ferryboat, and sought the bedside of his patient. Now, however, it was the turn of the islanders. The doctor had insisted upon his fee beforehand, and might do it again, when perhaps a guinea would be a scarcer commodity. He must be taught a lesson. There was but one ferryboat for Tory Island, as there was but one doctor. The ferryman, aware of all the circumstances, asked for his fee beforehand. He demanded not one guinea, but two. In vain the doctor argued, expostulated, threatened, and cajoled. The man of the oars was as obdurate as the man o pills. Two guineas, money down, or the doctor must stay where he was. The money was of course paid, and the doctor returned to his native Erin much impressed by the keen eye for business displayed by the natives of Tory Island.

**Tea.**

In spite of all the bad things that have been said against tea by medical men and others, John Chinaman's favourite fluid still remains popular, both with the classes and the masses. Dr. Thorne, who was Anthony Trollope's beau-ideal of a medical man, as he is the present writer's, was a perfect sponge for tea. After a long round in the country he would think nothing of taking four or five large breakfast cupfuls; and even then he would fain have persuaded his neice and housekeeper, Mary, to make a fresh brew. He never seemed to be one penny the worse for all such rash indulgence. Tea is undoubtedly the drink of drinks, after water. It is light and pleasant to the taste, and its gently-stimulating properties are beyond praise. Of course there is tea and tea. Some people present themselves and their friends with mere "wash." Tea need not be very strong; indeed, it is better not too strong. Everything depends on the quality of the leaf and the method of brewing. A famous physician of the present day used to go in for "five miuutes" tea—that is, tea brewed for five minutes only. The other day, the wife of a distinguished Q.C. informed the present writer that he had given up his five minutes' brew, and now prefers three minutes. Whether five or three minutes be better depends, perhaps, to some extent on the particular leaf which may happen to be used. It is certain, however, that tea should not be brewed too long if it is to be of the most perfect flavour. Cream is a very decided acquisition to tea. One wonders that the prosperous classes ever take tea without it. Cream is much more essential with tea than currant jelly is with mutton, or good looks in one's wife. It is no doubt quite possible to take too much tea, especially, as the Scotchman said of the whisky, if the tea be bad. But most people can enjoy and digest a fair quantity of tea once a-day, and some people, twice. There is one large hospital in London where, it is said, some of the lady nurses make tea for themselves (of course, at their own expense) eight or ten times a-day. That is a great deal too often; and nurses, who are half doctors, ought to know better. But once or twice a day, good tea, well brewed and judiciously tempered with cream and a little sugar, is a fluid, not only fit for the gods, but fit for the men and women of the nineteenth century.

**Wet Feet.**

In Scotland, where the children of the poor run about with bare feet, and often bare heads, colds and coughs are not nearly so common as in London, where both head and feet are clothed. People in the habit of arriving at conclusions *per saltum* may thence conclude that it is a foolish practice to wear boots. It is not a foolish practice to wear boots unless they are foolish boots. The foot clothing of women and children in London is often the very opposite of what it should be. In the recent damp weather, for example, many women and children have been going about with boots only fit for the driest part of summer. Wet feet, when encased in damp boots, are a very fruitful source of sore throat, and also of that peculiar form of laryngitis known as "false croup." In damp weather, even more than in cold weather, thick waterproof boots should be worn; and when outdoor work is over, the boots, and if need be the stockings also, should be promptly changed. City men, as well, who often get wet early in the day, should have additional pairs of boots at their offices, in order to prevent themselves sitting all day with damp feet. A little thought and a little trouble often make all the difference between sound health and chronic incapacity.

